# ERLIN2 promotes breast cancer cell survival by modulating endoplasmic reticulum stress pathways

**DOI:** 10.1186/1471-2407-12-225

**Published:** 2012-06-08

**Authors:** Guohui Wang, Gang Liu, Xiaogang Wang, Seema Sethi, Rouba Ali-Fehmi, Judith Abrams, Ze Zheng, Kezhong Zhang, Stephen Ethier, Zeng-Quan Yang

**Affiliations:** 1 Karmanos Cancer Institute, Wayne State University, Detroit, MI, 48201, USA; 2 Center for Molecular Medicine and Genetics, Wayne State University, Detroit, MI, 48201, USA; 3 Department of Oncology, Wayne State University, Detroit, MI, 48201, USA; 4 Department of Pathology, Wayne State University, Detroit, MI, 48201, USA; 5 Department of Immunology and Microbiology, Wayne State University, Detroit, MI, 48201, USA; 6 Biorepository Core, Wayne State University, Detroit, MI, 48201, USA; 7 Biostatistics Core of the Karmanos Cancer Institute, Wayne State University, Detroit, MI, 48201, USA; 8 Department of Medical Imaging and Interventional Radiology, State Key Laboratory of Oncology in South China, Cancer Center, Sun Yat-Sen University, No. 651, Dongfeng Road East, Guangzhou, 510060, China; 9 Department of Pathology and Laboratory Medicine, Hollings Cancer Center, Medical University of South Carolina, BEB 412, 68 President St, Charleston, SC 29425, USA

**Keywords:** Gene amplification, Breast cancer, Endoplasmic reticulum, ERLIN2

## Abstract

**Background:**

Amplification of the 8p11-12 region has been found in approximately 15% of human breast cancer and is associated with poor prognosis. Previous genomic analysis has led us to identify the *endoplasmic reticulum (ER) lipid raft-associated 2* (*ERLIN2*) gene as one of the candidate oncogenes within the 8p11-12 amplicon in human breast cancer, particularly in the luminal subtype. ERLIN2, an ER membrane protein, has recently been identified as a novel mediator of ER-associated degradation. Yet, the biological roles of ERLIN2 and molecular mechanisms by which ERLIN2 coordinates ER pathways in breast carcinogenesis remain unclear.

**Methods:**

We established the MCF10A-ERLIN2 cell line, which stably over expresses ERLIN2 in human nontransformed mammary epithelial cells (MCF10A) using the pLenti6/V5-ERLIN2 construct. ERLIN2 over expressing cells and their respective parental cell lines were assayed for *in vitro* transforming phenotypes. Next, we knocked down the ERLIN2 as well as the ER stress sensor IRE1α activity in the breast cancer cell lines to characterize the biological roles and molecular basis of the ERLIN2 in carcinogenesis. Finally, immunohistochemical staining was performed to detect ERLIN2 expression in normal and cancerous human breast tissues

**Results:**

We found that amplification of the *ERLIN2* gene and over expression of the ERLIN2 protein occurs in both luminal and Her2 subtypes of breast cancer. Gain- and loss-of-function approaches demonstrated that ERLIN2 is a novel oncogenic factor associated with the ER stress response pathway. The IRE1α/XBP1 axis in the ER stress pathway modulated expression of ERLIN2 protein levels in breast cancer cells. We also showed that over expression of ERLIN2 facilitated the adaptation of breast epithelial cells to ER stress by supporting cell growth and protecting the cells from ER stress-induced cell death.

**Conclusions:**

ERLIN2 may confer a selective growth advantage for breast cancer cells by facilitating a cytoprotective response to various cellular stresses associated with oncogenesis. The information provided here sheds new light on the mechanism of breast cancer malignancy

## Background

Breast cancer cells contain a large number of genetic alterations that act in concert to create the malignant phenotype. For example, the up-regulation of oncogenes, such as *Her2, c-MYC and CCND1,* directly contributes to the uncontrolled proliferation of breast cancer cells. For cancer cells to survive, they must acquire the ability to tolerate a series of oncogenesis-associated cellular stressors, which include DNA damage, proteotoxic-, mitotic-, metabolic-, and oxidative-stress [1,2]. However, very little is currently known about the genomic basis and molecular mechanisms that allow breast cancer cells to tolerate and adapt to these stresses. Amplification of 8p11-12 occurs in approximately 15% of human breast cancer (HBC). This region of amplification is significantly associated with disease-specific survival and distant recurrence in breast cancer patients [3-6]. Previous work in our laboratory, together with others, have identified the *endoplasmic reticulum (ER) lipid raft-associated 2* (*ERLIN2*, also known as *SPFH2C8ORF2*) gene as one of several candidate oncogenes within the 8p11-12 amplicon, based on statistical analysis of copy number increase and over expression [3,4,7]. Yet, the biological roles of ERLIN2 and molecular mechanisms by which ERLIN2 coordinates ER pathways in breast carcinogenesis remain unclear.

The ER is a cellular organelle primarily responsible for protein folding, lipid and sterol biosynthesis, and calcium storage. Physiological processes that increase protein folding demand or stimuli that disrupt the ER protein folding process can create an imbalance between ER protein folding load and capacity. This imbalance leads to the accumulation of unfolded or misfolded proteins in the ER: a condition referred to as “ER stress” [8,9]. The ER has evolved highly specific signaling pathways, collectively termed the “unfolded protein response” (UPR), to ensure protein folding fidelity and to protect the cell from ER stress. Upon activation of UPR, inositol-requiring protein 1 (IRE1α), the conserved ER stress sensor from yeasts to mammals, mediates splicing of the mRNA encoding X-box binding protein 1 (XBP1). XBP1 serves as a potent UPR *trans*-activator that helps protein refolding, transportation, and degradation in order to bolster ER capacity and facilitate cell adaptation to stress [8]. However, if UPR fails to restore ER homeostasis, ER stress-associated apoptosis will occur [10]. As part of the UPR program, ER-associated degradation (ERAD) targets aberrantly folded proteins in the ER. In addition to this “quality control” function, ERAD also accounts for the degradation of several metabolically-regulated ER proteins [11].

Recent studies provide evidence that UPR and ERAD components are highly expressed in various tumors, including human breast cancer [12-21]. During tumor development and progression, increased amounts of misfolded proteins caused by gene mutations, hypoxia, nutrient starvation, and high-levels of reactive oxygen species lead to ER stress [22,23]. The activation of UPR and ERAD induces an adaptive response in which the tumor cell attempts to overcome ER stress to facilitate cytoprotection. In this study, we demonstrated that amplification and the resultant over expression of ERLIN2 occurred in both luminal and Her2 subtypes of breast cancer. We also found that the UPR pathway, through the IRE1α/XBP1 axis, modulated the high-level expression of ERLIN2 protein. Furthermore, ERLIN2 had the ability to protect breast cancer cells from ER stress-induced cell death. Thus, ERLIN2 is a novel mediator of ER stress response and thus amplification and over expression of ERLIN2 may facilitate the adaptation of breast cancer cells to the various cellular stresses associated with oncogenesis.

## Materials and methods

### Cell lines and cell culture conditions

The culture conditions of of SUM breast cancer cells and the immortalized non-tumorigenic MCF10A cells are described in the Additional file 1: Materials and Methods.

### Genomic array CGH

Genomic array CGH experiments were performed using the Agilent 44 K human genome CGH microarray chip (Agilent Technologies, Palo Alto, CA). Agilent's CGH Analytics software was used to calculate various measurement parameters, including log2 ratio of total integrated Cy-5 and Cy-3 intensities for each probe.

### Semiquantitative RT-PCR reactions

Total RNA was prepared from human breast cancer cell lines and the MCF10A cell line by standard methods [3,24]. For RT-PCR reactions, RNA was converted into cDNA *via* a reverse transcription reaction using random hexamer primers. Primers were ordered from Invitrogen (Carlsbad, CA). A GAPDH primer set was used as a control. Semiquantitative RT-PCR was done using the iQSYBR Green Supermix (Bio-Rad, Hercules, CA).

### Lentivirus construction and transduction of cells

The lentiviral expression construct containing the *ERLIN2* gene (pLenti-ERLIN2), was established as previously described [3]. The lentivirus for pLenti-ERLIN2 was generated and used to infect the immortalized, nontransformed mammary epithelial MCF10A cells. Control infections with pLenti-LacZ virus were performed in parallel with the pLenti-ERLIN2 infections. Selection began 48 h after infection in growth medium with 10 μg/mL blasticidin in the absence of either insulin or epidermal growth factor (EGF). Upon confluence, selected cells were passaged and serially cultured.

### Three-dimensional morphogenesis assays in matrigel

For three-dimensional morphogenesis assays in Matrigel, cells grown in monolayer culture were detached by trypsin/EDTA treatment and seeded in Matrigel (BD Biosciences, San Jose, CA) precoated 8-well chamber slides. The appropriate volume of medium was added and cells were maintained in culture for 10–18 days. Phase-contrast images and immunostaining images were taken using bright-field and confocal microscopy.

### Lentivirus-mediated shRNA knockdown of gene expression

We knocked down the expression of the human *ERLIN2* gene in breast cancer cell lines and in the MCF10A cell line using the Expression Arrest GIPZ lentiviral shRNAmir system (OpenBiosystems, Huntsville, AL). Lentivirus was produced by transfecting 293FT cells with the combination of the lentiviral expression plasmid DNA and Trans-Lentiviral packaging mix (OpenBiosystems. Huntsville, AL). For cell infection, viral supernatants were supplemented with 6 μg/mL polybrene and incubated with cells for 24 hours. Cells expressing shRNA were selected with puromycin for 2–3 weeks for functional studies (cell proliferation and colony formation assays) and for 4 to 10 days after infection for RNA extraction.

### Recombinant adenoviral or retrovirus infection

Adenoviruse vectors for expressing flag-tagged IRE1α isoforms, including wild type IRE1α (Ad-IRE1α WT), IRE1α kinase mutant (Ad-IRE1α K599A), and IRE1α RNase mutant (Ad-IRE1α K907A), were kindly provided by Dr. Yong Liu (Institute for Nutritional Sciences, Shanghai, China) and amplified using the AdEasy System (Stratagene) [25,26]. Retrovirus expressing spliced XBP1 was kindly provided by Dr. Lauri Glimcher (Harvard University) [27]. For infection of cells with adenovirus and retrovirus, cells were seeded in six-well plates. After 24 h, cells were infected with adenovirus expressing wild type IRE1α (Ad-IRE1α WT), IRE1α kinase mutant (Ad-IRE1α K599A), IRE1α RNase mutant (Ad-IRE1α K907A), and retrovirus expressing spliced XBP1 as described previously [28,29].

### Tissue array and immunohistochemistry (IHC) staining

Human breast cancer tissue array was obtained from Nuclea Biotechnologies (Pittsfield, MA). Immunohistochemistry was performed on tumor tissue sections using the standard laboratory protocols [30]. Briefly, after deparaffinizing and hydrating with phosphate-buffered saline (PBS) buffer (pH 7.4), the sections were pretreated with hydrogen peroxide (3%) for 10 minutes to remove endogenous peroxidase, followed by antigen retrieval *via* steam bath for 20 minutes in EDTA. A primary antibody was applied, followed by washing and incubation with the biotinylated secondary antibody for 30 minutes at room temperature. Detection was performed with diaminobenzidine (DAB) and counterstaining with Mayer hematoxylin followed by dehydration and mounting. Immunostained slides were blindly evaluated under a transmission light microscope. Areas of highest staining density were identified for evaluating the expression in tumors.

## Results

### ERLIN2 is amplified and over expressed in human breast cancer cells

**Figure 1:**
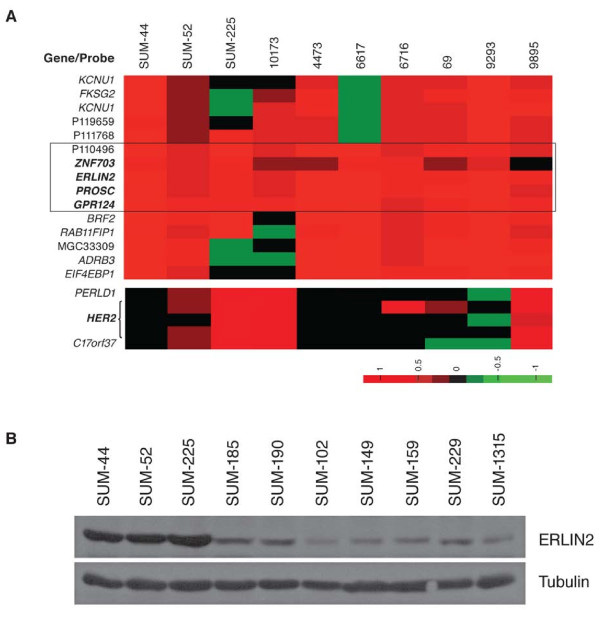
**(a) Genomic copy number profiles of the ERLIN2 region analyzed on the Agilent oligonucleotide array CGH in 3 SUM breast cancer cell lines and 7 primary breast cancer specimens**. Tumors are displayed vertically and array probes are displayed horizontally by genome position. Log2 ratio in a single sample is relative to normal female DNA and is depicted according to the color scale (bottom). Red indicates relative copy number gain, whereas green indicates relative copy number loss. (**b**) ERLIN2 protein levels were analyzed by Western blot in ten breast cancer cell lines with or without *ERLIN2* amplification.

Recently, we used quantitative genomic PCR and array comparative genomic hybridization (CGH) to profile copy number alterations in 10 human breast cancer cell lines and 90 primary human breast cancers [3,6,31]. Analysis of our array CGH data showed that ERLIN2 gene was commonly amplified in 30% of the cell lines tested, as well as in 7.8% of breast cancer specimens tested (Figure 1a). Previously, we and several other laboratories have demonstrated that the 8p11-12 amplicon occurs mainly in the luminal subtype of breast cancer cells, such as the SUM-44 and SUM-52 cell lines. However, SUM-225 is a *Her2*-amplified HBC cell line [31,32]. We also found two primary tumors, 10173 and 9895, which have *Her2* gene amplifications in addition to the amplification of the ERLIN2 gene (Figure 1a). To obtain further support for a potential involvement of the ERLIN2 region in breast cancer, we searched the published database of the Affymetrix 250 K array CGH [33]. We found that 42 of the 243 HBC lines and primary samples in the array exhibited amplification of the ERLIN2 region. Interestingly, eight of the *ERLIN2*-amplified samples showed co-amplification of the *Her2* gene (Additional file 1: Figure S 1). Next, we measured ERLIN2 protein levels in ten breast cancer cell lines by Western blot analysis. In correlation with *ERLIN2* gene amplification, ERLIN2 protein levels in SUM-44, SUM-52, and SUM-225 cells were dramatically greater than the levels in breast cancer cell lines without *ERLIN2* gene amplification (Figure 1b). The presence of the ERLIN2 amplification in both luminal and Her2 subtypes of breast cancer prompted us to further investigate the role of the *ERLIN2* gene in breast cancer progression.

### ERLIN2 plays a functional role in breast cancer cells

**Figure 2:**
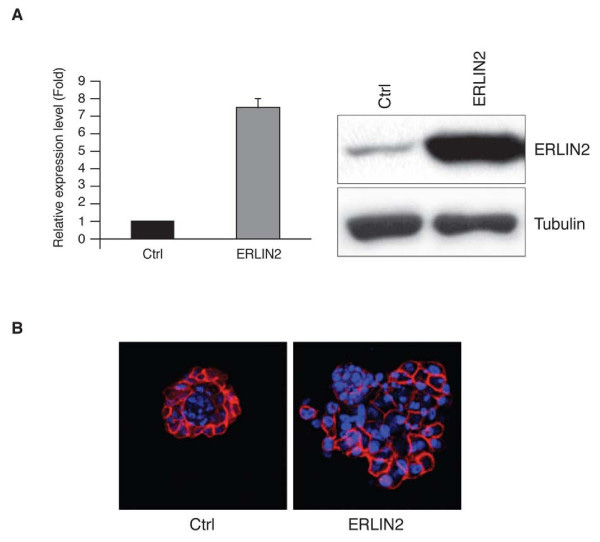
**(a) Stable overexpressing ERLIN2 in MCF10A cells (MCF10A-ERLIN2) with the pLenti6/V5-ERLIN2 construct**. Over expression of ERLIN2 mRNA and protein in this cell line was confirmed with semi-quantitative RT-PCR (right panel) and western blot assays (left panel). (**b**) Effects of ERLIN2 on mammary acinar morphogenesis. MCF10A-ERLIN2 and control cells were cultured on a bed of Matrigel. Representative images show the structures with staining for actin with phalloidin conjugated to Alexa Fluor-568 (red), and DAPI as a marker of nuclei (blue).

Next, we addressed whether *ERLIN2* possess transforming properties. We transduced the immortalized, nontransformed mammary epithelial cell line, MCF10A, with lentivirus expressing ERLIN2 or control LacZ. Semi-quantitative RT-PCR (qRT-PCR), Western blot and immunofluorescence staining confirmed the over expression of ERLIN2 protein in MCF10A-ERLIN2 cells (Figure 2a and Additional file 1: Figure S 2). The infected MCF10A cells were then subjected to analyses for growth rates, growth factor-independent proliferation, anchorage-independent growth, and three-dimensional morphogenesis assays. Growth curves and colony formation assays in MCF10A cells showed that forced expression of ERLIN2 resulted in growth factor-independent proliferation in insulin-like growth factor-deficient media. To further examine the effects of ERLIN2 in a context that more closely resembles *in vivo* mammary architecture, we assessed the consequences of ERLIN2 over expression on three-dimensional morphogenesis in Matrigel. Although MCF10A cells formed polarized, growth-arrested acinar structures with hollow lumens similar to the glandular architecture *in vivo*, MCF10A-ERLIN2 cells formed abnormal acini at a high frequency that was grossly disorganized, and contained filled lumens (Figure 2b).

**Figure 3:**
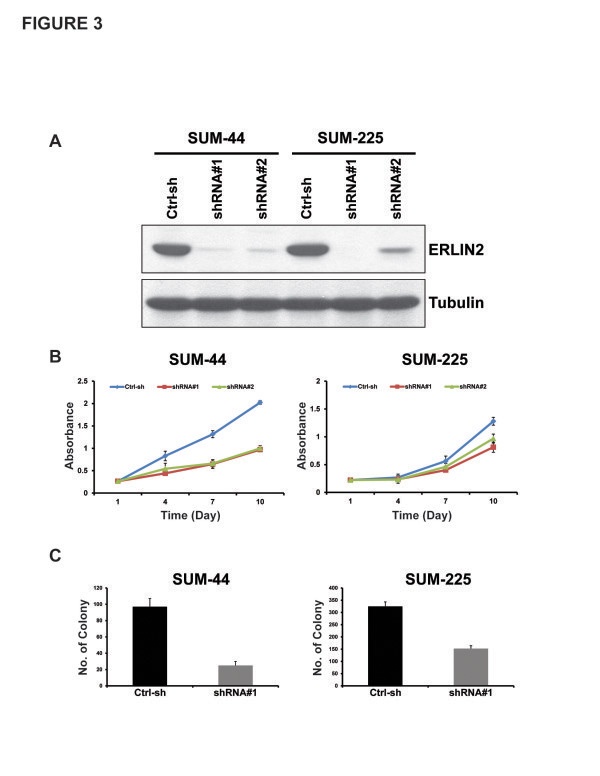
**shRNA-mediated knockdown of ERLIN2 inhibits monolayer and anchorage-independent cell growth in breast cancer cells with ERLIN2 amplification.** (**a**) Knockdown of ERLIN2 expression in SUM-44 and SUM-225 cells with two different shRNAs was confirmed by western blot. (**b**) *In vitro* growth rate of SUM-44 and SUM-225 cells with ERLIN2 shRNA treatment compared to cells with control shRNA treatment. (**c**) Knockdown of ERLIN2 reduces colony formation in soft agar. SUM-44 and SUM-225 cells were tranfected with ERLIN2 shRNA#1 or control shRNA. The colony numbers were counted 3 weeks later (P < 0.05).

To further explore the pathophysiological function of ERLIN2 over expression, we stably silenced the *ERLIN2* gene in SUM-44 and SUM-225 breast cancer cells using the lentiviral-based shRNA system. To perform RNAi knockdown experiments, we utilized pGIPZ-ERLIN2 shRNA expression constructs in which TurboGFP and shRNA were part of a bicistronic transcript allowing for the visual marking of the shRNA-expressing stable cells. qRT-PCR and Western blot analysis indicated a marked reduction in expression levels of ERLIN2 mRNA and protein in the stable ERLIN2-shRNA-transduced SUM-44 and SUM-225 cell lines as compared with the control cell lines infected with a non-silencing shRNA lentiviral control (Figure 3a). Among the two targeted vectors used, ERLIN2-shRNA vector #1 produced a more striking knockdown effect: infected SUM-225 cells had a nearly complete loss of ERLIN2 protein expression (Figure 3a). We did not detect any change in ERLIN1 mRNA and protein levels in ERLIN2-shRNA knockdown cells, thus ruling out the possiblity of off-target effects by ERLIN2-shRNAs (Data not shown). Cell growth and proliferation analyses showed that knockdown of ERLIN2 slowed the proliferation rate of SUM-44 and SUM-225 cells, but had only a minor effect on SUM-102 and MCF10A cells, which lack ERLIN2 amplification (Figure 3b). Importantly, knockdown of ERLIN2 in SUM-44 and SUM-225 cells also suppressed anchorage-independent growth in soft agar, one of the hallmark characteristics of aggressive cancer cells. (Figure 3c). Taken together, results from over expression and knockdown experiments suggested *ERLIN2* plays a role in cell proliferation and maintenance of transforming phenotypes in breast cancer cells with the 8p11-12 amplification.

### Expression of ERLIN2 is regulated by the ER pathway through IRE1α/XBP1

**Figure 4:**
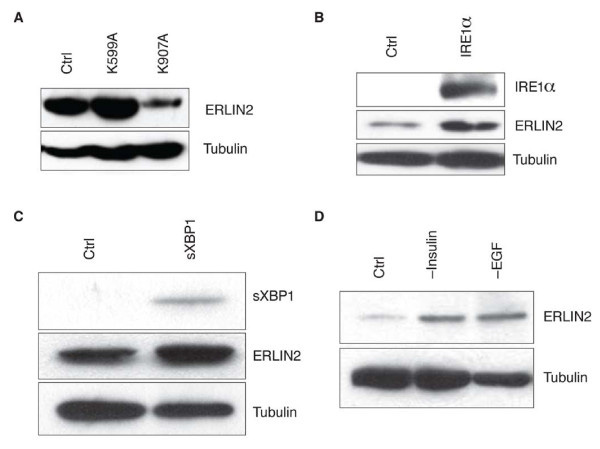
**(a) The knockdown of the IRE1α RNase activity (K907A) reduced levels of ERLIN2 protein in SUM-44 cells.** Forced expression of wild-type IRE1α (**b**) and its substrate, spliced XBP1 (**c**), leads to increased expression of ERLIN2 at protein level in MCF10A cells. (**d**) ERLIN2 expression in MCF10A cells was analyzed by western blot after culture 48 hours in insulin- or EGF-depleted media, compared to that in normal culture media.

Recent studies have identified ERLIN2 both as a novel component of lipid raft domains in the ER membrane and as a substrate recognition factor during ERAD of activated inositol triphosphate receptors (IP3R) as well as other substrates [34-36]. IRE1α is the primary ER stress sensor implicated in the regulation of the ERAD pathway [37]. Under ER stress, IRE1α undergoes auto-phosphorylation to activate its RNase activity, which triggers one of the UPR cascades through splicing *Xbp1* mRNA [8]. Previous work has demonstrated that breast cancer cells over express XBP1 [38,39], while we observed that SUM-44, SUM-52 and SUM-225 cell lines over expressed total and activated XBP1 (Additional file 1: Figure S 3 Additional file 2: Table S 1). To evaluate the possibility of an association between ERLIN2 expression and the IRE1α-mediated UPR pathway in HBC, we inhibited IRE1α RNase or kinase activity in breast cancer cells. To accomplish this, we used adenoviral-based expression system to introduce the previously characterized IRE1 kinase dominant-negative mutant (IRE1 K599A) or the IRE1 RNase dominant-negative mutant (IRE1 K907A) into breast cancer cells [26,40,41]. We chose SUM-44 cells for this experiment because the SUM-44 cells are very amenable to adenovirus-mediated expression. The inhibition of the IRE1α RNase activity significantly reduced protein levels of ERLIN2 in SUM-44 cells (Figure 4a). In addition, forcible expression of wild-type IRE1α or spliced XBP1 in MCF10A cells resulted in increased expression levels of endogenous ERLIN2 protein (Figure 4b and c). However, quantitative real-time RT-PCR analysis showed that over expression of IRE1α or spliced XBP1 did not increase expression of the *ERLIN2* mRNA (Data not shown). Next, we asked whether ERLIN2 expression was induced by stress inducers in normal mammary epithelial cells. Our group routinely cultures MCF10A cells in serum-free, growth factor-supplemented media. Oncogenesis-associated conditions, such as nutrient or growth factor depletion, can cause pathophysiologic ER stress [22,23]. When MCF10A cells were cultured in media lacking insulin or EGF, expression levels of endogenous ERLIN2 protein in MCF10A were increased as compared with levels in cells cultured in normal media (Figure 4d). Our observations suggest that the ER stress pathway likely regulates ERLIN2 protein expression through IRE1α-actived XBP1 in human breast epithelial cells.

### ERLIN2 promotes breast cancer cell survival

**Figure 5:**
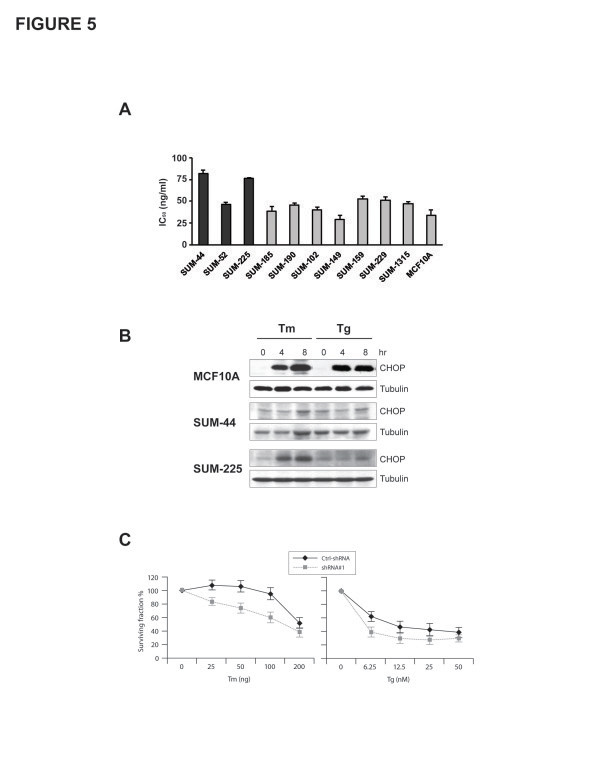
**(a) IC**_**50**_**values for the ER stress-inducing reagent Tm, in ten breast cancer cell lines as well as in the MCF10A cells (b) The expression level of CHOP in SUM-225, SUM-44 breast cancer cells and MCF10A control cells was analyzed with Western blot after Tm (500 ng) or Tg (400 nM) treatment.** (**c**) Cell viability of ERLIN2 knockdown and control SUM-225 cells was measured with MTT assays after exposure to different concentrations of the Tm or Tg for 72 hours.

Next, we tested if amplification and over expression of ERLIN2 enhances the resistance to a variety of stressors to promote cancer cell survival. Figure 5a shows the IC_50_ values for the ER stress-inducing reagent Tunicamycin (Tm), in ten breast cancer cell lines as well as in the nontransformed human mammary epithelial cell line MCF10A. SUM-44 and SUM-225 cells, which have ERLIN2 amplification, had significantly higher TM IC_50_ values than cell lines without ERLIN2 amplification (P < 0.05). We obtained similar results with Thapsigargin (Tg) treatment of SUM-225 cells (data not shown). Expression of the CCAAT/enhancer-binding protein (C/EBP) homology protein (CHOP) is characteristic of the ER stress–mediated apoptotic pathway. In response to treatment with Tm or Tg, expression of CHOP dramatically increased in control MCF10A cells (Figure 5b). However, induction of CHOP by Tm and Tg treatment was weaker or barely detectable in SUM-44 and SUM-225 cells (Figure 5b). Next, to determine whether suppressing ERLIN2 in breast cancer cells re-sensitize them to ER-stress, we challenged stable ERLIN2-knockdown SUM-44 and SUM-225 cells with Tm and Tg for 72 hours and evaluated their viability using the MTT assay. Knockdown of ERLIN2 resulted in increased sensitivity to Tm or Tg -induced cell death (Figure 5c). Our data suggested that over expression of ERLIN2 may facilitate the adaptation of breast epithelial cells to ER stress by supporting cell growth. Future investigations are required to more precisely address the mechanism by which ERLIN2 promotes breast cancer cell survival.

**Figure 6:**
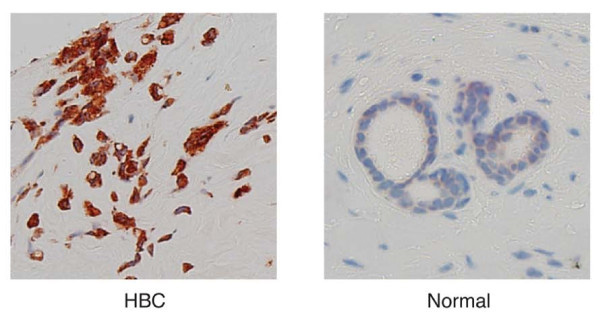
Immunohistotochemical staining of ERLIN2 protein on a representative HBC sample and a normal control.

### Expression of ERLIN2 in breast tissues: Carcinomas and normal

We evaluated the expression of ERLIN2 in normal and cancerous human breast tissues using immunohisyochemistry (IHC) in breast cancer tissue arrays. We first confirmed the specificity and sensitivity of the ERLIN2 antibody for visualizing ERLIN2 expression in formalin-fixed, paraffin-embedded breast cancer cell lines. Consistent with the immunoblotting data, SUM-225 cells displayed significantly higher levels of positive staining as compared with the MCF10A control cells (Additional file 1: Figure S 4). The tissue array included 34 breast carcinomas and 17 normal breast tissue, which included 14 cases of adjacent normal counterparts. ERLIN2 expression was scored based on the staining intensity: 0 (negative), 1 + (weak), 2 + (low); 3+ (moderate) or 4 + (strong). In breast carcinomas samples, 11 (32.4%) stained ERLIN2 strongly and 13 (38.2%) moderately (Figure 5, Additional file 1: Figure S 5 Additional file 2: Table S 2). In contrast, no strong or moderate staining was observed in the 17 normal breast tissues. The staining intensities of ERLIN2 were significantly higher in tumor cells than in normal tissue cells (P = 0.001).

## Discussion

The 8p11-12 amplicon in HBC has been the subject of a number of studies using high-resolution genomic analysis of copy number and gene expression [3-6,42,43]. We previously found that the 8p11-12 amplicon has a highly complex genomic structure and that the size of the amplicon in three HBC lines, SUM-44, SUM-52 and SUM-225, is highly variable [6,31]. Moreover, the sub-amplicon of 8p11-12 that carries the *ERLIN2* gene amplification was more frequently identified in HBCs [4,7]. Previous studies have demonstrated that the 8p11-12 amplicon occurs mainly in the luminal subset of breast cancer cells, such as SUM-44 cells, a subset that also expresses the estrogen receptor [3,4,7,44-46]. Here we report that the co-amplification of the *ERLIN2* region occurred in a subset of *HER2*-amplified breast cancer cells, including SUM-225 cells. Our recent studies with Her2 model cells demonstrated that over expression of Her2 alone is not sufficient to induce full transformation *in vitro* and is not tumorigenic *in vivo*[47]. In contrast, *Her2*-amplified SUM225 breast cancer cells are fully transformed *in vitro* and tumorgic *in vivo*[48]*.* In this study, *in vitro* transforming and shRNA assays provided evidence that ERLIN2 is the most likely non- classical oncogene within this 8p11-12 minimal common amplified region. Our results suggest that the *ERLIN2* plays a role in cell proliferation and maintenance of transforming phenotypes in breast cancer cells with the 8p11-12 amplification.

ERLIN2 belongs to a larger family of proteins that share an evolutionarily conserved stomatin/prohibitin/flotillin/HflK/C (SPFH) domain. SPFH-containing proteins localize to different membranes, but have common characteristics. For example, N-terminal sequences are required for subcellular localization and membrane attachment, while the coiled-coil motifs located at the C-terminal side of SPFH domain mediate the assembly of high-molecular-weight complexes [49]. ERLIN2 and its homologue ERLIN1 were originally identified as components of lipid rafts that localize to the ER [36]. More recently, ERLIN2 has been recognized as a novel mediator of ERAD [34-36,50]. ERLIN2 binds to activated IP3Rs and other ERAD substrates, leading to polyubiquitination and subsequent degradation of these substrates [34,35].

Of particular interest in this study, we found that the UPR pathway modulated ERLIN2 protein expression in breast cancer cells through the IRE1α/XBP1 axis. Forced expression of IRE1α, or spliced XBP1, the target of IRE1α under ER stress, up-regulated expression of the ERLIN2 protein, while knockdown of IRE1α RNase activity decreased ERLIN2 expression in the *ERLIN2*-amplified breast cancer cells. These gain- and loss-of-function studies provided support that the IRE1α/XBP1-mediated UPR pathway in HBC regulated production of ERLIN2. Importantly, our study also showed that the depletion of nutrient and growth signals, a condition that is associated with oncogenesis and ER stress, can increase ERLIN2 production in breast epithelial cells. However, over expression of IRE1α or spliced XBP1 did not increase expression of the *ERLIN2* mRNA level, suggesting regulation at the post-transcription level. In the present study, we also showed that expression of primary breast cancer cells significantly up regulated ERLIN2 protein expression as compared with normal breast cells. As we had described earlier, amplification of the *ERLIN2* gene, as part of the 8p11-12 amplicon, occurs in approximately 15% of human breast cancer. It is reported that XBP1 is over expressed in aggressive breast cancer and associated with cancer cell survival and therapy resistance [51]. In the ten SUM breast cancer cell lines we investigated, three lines have both ERLIN2 gene amplification and up-regulation of activated XBP1, resulting in dramatically high-level expression of ERLIN2 protein. In contrast, two lines with up-regulation of the XBP1, but no ERLIN2 gene amplification, had moderately high-expression of the ERLIN2 protein. Taken together, our results raise an intriguing notion that the breast cancer cells may utilize gene amplification and the UPR pathway to regulate ERLIN2 protein over-production under oncogenic stress conditions.

In response to ER stress, cells activate UPR to reprogram gene transcription and translation. Depending on the type and/or degree of the stress, cells can differentially activate the UPR pathways in order to make survival or death decisions [52]. The literature indicates that the UPR branch, through IRE1α/XBP1, plays a critical role in cell adaptation to ER stress by increasing protein refolding and degradation of misfolded proteins, and by bolstering the protein-folding capacity and secretion potential of the ER [20,52,53]. Cancer cells may adapt to the cellular stress and evade stress-induced apoptotic pathways by differentially activating the UPR branches. Indeed, tumor microenvironment has been characterized by a ‘baseline’ level of ER stress response that promotes tumor development and metastasis [20].

## Conclusions

In the present study, we show that over expression of ERLIN2 may facilitate the adaptation of breast epithelial cells to ER stress by supporting cell growth and protecting the cells from ER stress-induced apoptosis. These results suggest that ERLIN2 confers a selective growth advantage for breast cancer cells by facilitating a cytoprotective response to various cellular stresses associated with oncogenesis. The information provided here sheds new light the mechanism of breast cancer malignancy.

## Abbreviations

Her2, v-erb-b2 erythroblastic leukemia viral oncogene homolog 2 neuro/glioblastoma derived oncogene homolog (avian); c-MYC, v-myc myelocytomatosis viral oncogene homolog (avian); CCND1, cyclin D1; HBC, Human breast cancer; ER, Endoplasmic reticulum; ERLIN2, Endoplasmic reticulum lipid raft-associated 2; UPR, Unfolded protein response; IRE1, Inositol-requiring protein 1; XBP1, X-box binding protein 1; ERAD, ER-associated degradation; CGH, Comparative genomic hybridization; shRNA, Short hairpin RNA; IP3R, Inositol triphosphate receptors; Tm, Tunicamycin; Tg, Thapsigargin; CHOP, The CCAAT/enhancer-binding protein (C/EBP) homology protein; IHC, Immunohisyochemistry.

## Competing interests

The authors declare that they have no competing interests.

## Authors' contributions

GHW, GL and XGW performed most of the experiments, participated in designing the study, analyzing the data. SS, RAF and ZZ were involved in IHC staining experiments. SE participated in design of the study. KZZ and ZQY conceived, coordinated, designed and procured funding for the study and wrote the manuscript. All authors gave final approval for the manuscript to be published.

## Pre-publication history

The pre-publication history for this paper can be accessed here:

http://www.biomedcentral.com/1471-2407/12/225/prepub

## Supplementary Material

Additional file 1 **Materials and Methods**[54-58]**.** Click here for file

Additional file 2: Table S1 Expression Levels of XBP1, ERLIN1 and ERLIN2 in Ten SUM BreastCancer Cell Lines Using Our Affymetrix Array Database. Table S2: Expression of ERLIN2 in breast tissues:carcinomas and normal. Figure S1. Figure S2. Figure S3. Figure S4. Figure S5. Click here for file

## References

[B1] Luo J Solimini NL Elledge SJ Principles of cancer therapy: oncogene and non-oncogene addiction Cell 2009 136 5 823 837 10.1016/j.cell.2009.02.024 19269363 PMC2894612

[B2] Solimini NL Luo J Elledge SJ Non-oncogene addiction and the stress phenotype of cancer cells Cell 2007 130 6 986 988 10.1016/j.cell.2007.09.007 17889643

[B3] Yang ZQ Streicher KL Ray ME Abrams J Ethier SP Multiple interacting oncogenes on the 8p11-p12 amplicon in human breast cancer Cancer Res 2006 66 24 11632 11643 10.1158/0008-5472.CAN-06-2946 17178857

[B4] Gelsi-Boyer V Orsetti B Cervera N Finetti P Sircoulomb F Rouge C Lasorsa L Letessier A Ginestier C Monville F Comprehensive profiling of 8p11-12 amplification in breast cancer Molecular cancer research: MCR 2005 3 12 655 667 10.1158/1541-7786.MCR-05-0128 16380503

[B5] Garcia MJ Pole JC Chin SF Teschendorff A Naderi A Ozdag H Vias M Kranjac T Subkhankulova T Paish C A 1 Mb minimal amplicon at 8p11-12 in breast cancer identifies new candidate oncogenes Oncogene 2005 24 33 5235 5245 10.1038/sj.onc.1208741 15897872

[B6] Yang ZQ Albertson D Ethier SP Genomic organization of the 8p11-p12 amplicon in three breast cancer cell lines Cancer Genet Cytogenet 2004 155 1 57 62 10.1016/j.cancergencyto.2004.03.013 15527903

[B7] Kwek SS Roy R Zhou H Climent J Martinez-Climent JA Fridlyand J Albertson DG Co-amplified genes at 8p12 and 11q13 in breast tumors cooperate with two major pathways in oncogenesis Oncogene 2009 10.1038/onc.2009.34 PMC2722962 19330026

[B8] Ron D Walter P Signal integration in the endoplasmic reticulum unfolded protein response Nat Rev Mol Cell Biol 2007 8 7 519 529 10.1038/nrm2199 17565364

[B9] Zhang K Kaufman RJ From endoplasmic-reticulum stress to the inflammatory response Nature 2008 454 7203 455 462 10.1038/nature07203 18650916 PMC2727659

[B10] Zhang K Kaufman RJ Identification and characterization of endoplasmic reticulum stress-induced apoptosis in vivo Methods Enzymol 2008 442 395 419 18662581 10.1016/S0076-6879(08)01420-1 PMC2865177

[B11] Schroder M Kaufman RJ ER stress and the unfolded protein response Mutat Res 2005 569 1–2 29 63 15603751 10.1016/j.mrfmmm.2004.06.056

[B12] Dong D Ni M Li J Xiong S Ye W Virrey JJ Mao C Ye R Wang M Pen L Critical role of the stress chaperone GRP78/BiP in tumor proliferation, survival, and tumor angiogenesis in transgene-induced mammary tumor development Cancer Res 2008 68 2 498 505 10.1158/0008-5472.CAN-07-2950 18199545

[B13] Pyrko P Schonthal AH Hofman FM Chen TC Lee AS The unfolded protein response regulator GRP78/BiP as a novel target for increasing chemosensitivity in malignant gliomas Cancer Res 2007 67 20 9809 9816 10.1158/0008-5472.CAN-07-0625 17942911

[B14] Daneshmand S Quek ML Lin E Lee C Cote RJ Hawes D Cai J Groshen S Lieskovsky G Skinner DG Glucose-regulated protein GRP78 is up-regulated in prostate cancer and correlates with recurrence and survival Hum Pathol 2007 38 10 1547 1552 10.1016/j.humpath.2007.03.014 17640713

[B15] Fu Y Li J Lee AS GRP78/BiP inhibits endoplasmic reticulum BIK and protects human breast cancer cells against estrogen starvation-induced apoptosis Cancer Res 2007 67 8 3734 3740 10.1158/0008-5472.CAN-06-4594 17440086

[B16] Hetz C The UPR as a survival factor of cancer cells: More than folding proteins? Leuk Res 2009 10.1016/j.leukres.2009.02.017 19285722

[B17] Ran Y Hu H Hu D Zhou Z Sun Y Yu L Sun L Pan J Liu J Liu T Derlin-1 is overexpressed on the tumor cell surface and enables antibody-mediated tumor targeting therapy Clin Cancer Res 2008 14 20 6538 6545 10.1158/1078-0432.CCR-08-0476 18927294

[B18] Virrey JJ Dong D Stiles C Patterson JB Pen L Ni M Schonthal AH Chen TC Hofman FM Lee AS Stress chaperone GRP78/BiP confers chemoresistance to tumor-associated endothelial cells Molecular cancer research: MCR 2008 6 8 1268 1275 10.1158/1541-7786.MCR-08-0060 18708359 PMC2593417

[B19] Moenner M Pluquet O Bouchecareilh M Chevet E Integrated endoplasmic reticulum stress responses in cancer Cancer Res 2007 67 22 10631 10634 10.1158/0008-5472.CAN-07-1705 18006802

[B20] Wang G Yang ZQ Zhang K Endoplasmic reticulum stress response in cancer: molecular mechanism and therapeutic potential Am J Transl Res 2010 2 1 65 74 20182583 PMC2826823

[B21] Tsai YC Weissman AM The Unfolded Protein Response, Degradation from Endoplasmic Reticulum and Cancer Genes Cancer 2010 1 7 764 778 10.1177/1947601910383011 21331300 PMC3039444

[B22] Healy SJ Gorman AM Mousavi-Shafaei P Gupta S Samali A Targeting the endoplasmic reticulum-stress response as an anticancer strategy Eur J Pharmacol 2009 625 1–3 234 246 19835867 10.1016/j.ejphar.2009.06.064

[B23] Rutkowski DT Hegde RS Regulation of basal cellular physiology by the homeostatic unfolded protein response J Cell Biol 2010 189 5 783 794 10.1083/jcb.201003138 20513765 PMC2878945

[B24] Yang ZQ Imoto I Fukuda Y Pimkhaokham A Shimada Y Imamura M Sugano S Nakamura Y Inazawa J Identification of a novel gene, GASC1, within an amplicon at 9p23-24 frequently detected in esophageal cancer cell lines Cancer Res 2000 60 17 4735 4739 10987278

[B25] Qiu Y Mao T Zhang Y Shao M You J Ding Q Chen Y Wu D Xie D Lin X A crucial role for RACK1 in the regulation of glucose-stimulated IRE1alpha activation in pancreatic beta cells Sci Signal 3 106 ra7 20103773 10.1126/scisignal.2000514 PMC2940714

[B26] Tirasophon W Lee K Callaghan B Welihinda A Kaufman RJ The endoribonuclease activity of mammalian IRE1 autoregulates its mRNA and is required for the unfolded protein response Genes Dev 2000 14 21 2725 2736 10.1101/gad.839400 11069889 PMC317029

[B27] Iwakoshi NN Lee AH Vallabhajosyula P Otipoby KL Rajewsky K Glimcher LH Plasma cell differentiation and the unfolded protein response intersect at the transcription factor XBP-1 Nat Immunol 2003 4 4 321 329 12612580 10.1038/ni907

[B28] Zhang K Wang S Malhotra J Hassler JR Back SH Wang G Chang L Xu W Miao H Leonardi R The unfolded protein response transducer IRE1alpha prevents ER stress-induced hepatic steatosis EMBO J 2011 30 7 1357 1375 10.1038/emboj.2011.52 21407177 PMC3094110

[B29] Yang ZQ Moffa AB Haddad R Streicher KL Ethier SP Transforming properties of TC-1 in human breast cancer: interaction with FGFR2 and beta-catenin signaling pathways Int J Cancer 2007 121 6 1265 1273 10.1002/ijc.22831 17520678

[B30] Ali-Fehmi R Che M Khalifeh I Malone JM Morris R Lawrence WD Munkarah AR The effect of cyclooxygenase-2 expression on tumor vascularity in advanced stage ovarian serous carcinoma Cancer 2003 98 7 1423 1429 10.1002/cncr.11650 14508829

[B31] Ray ME Yang ZQ Albertson D Kleer CG Washburn JG Macoska JA Ethier SP Genomic and expression analysis of the 8p11-12 amplicon in human breast cancer cell lines Cancer Res 2004 64 1 40 47 10.1158/0008-5472.CAN-03-1022 14729606

[B32] Forozan F Veldman R Ammerman CA Parsa NZ Kallioniemi A Kallioniemi OP Ethier SP Molecular cytogenetic analysis of 11 new breast cancer cell lines Br J Cancer 1999 81 8 1328 1334 10.1038/sj.bjc.6695007 10604729 PMC2362964

[B33] Beroukhim R Mermel CH Porter D Wei G Raychaudhuri S Donovan J Barretina J Boehm JS Dobson J Urashima M The landscape of somatic copy-number alteration across human cancers Nature 2010 463 7283 899 905 10.1038/nature08822 20164920 PMC2826709

[B34] Pearce MM Wang Y Kelley GG Wojcikiewicz RJ SPFH2 mediates the endoplasmic reticulum-associated degradation of inositol 1,4,5-trisphosphate receptors and other substrates in mammalian cells J Biol Chem 2007 282 28 20104 20115 10.1074/jbc.M701862200 17502376

[B35] Pearce MM Wormer DB Wilkens S Wojcikiewicz RJ An ER membrane complex composed of SPFH1 and SPFH2 mediates the ER-associated degradation of IP3 receptors J Biol Chem 2009 10.1074/jbc.M809801200 PMC2667730 19240031

[B36] Browman DT Resek ME Zajchowski LD Robbins SM Erlin-1 and erlin-2 are novel members of the prohibitin family of proteins that define lipid-raft-like domains of the ER J Cell Sci 2006 119 Pt 15 3149 3160 16835267 10.1242/jcs.03060

[B37] Yoshida H Matsui T Hosokawa N Kaufman RJ Nagata K Mori K A time-dependent phase shift in the mammalian unfolded protein response Dev Cell 2003 4 2 265 271 10.1016/S1534-5807(03)00022-4 12586069

[B38] Fujimoto T Onda M Nagai H Nagahata T Ogawa K Emi M Upregulation and overexpression of human X-box binding protein 1 (hXBP-1) gene in primary breast cancers Breast Cancer 2003 10 4 301 306 10.1007/BF02967649 14634507

[B39] Davies MP Barraclough DL Stewart C Joyce KA Eccles RM Barraclough R Rudland PS Sibson DR Expression and splicing of the unfolded protein response gene XBP-1 are significantly associated with clinical outcome of endocrine-treated breast cancer Int J Cancer 2008 123 1 85 88 10.1002/ijc.23479 18386815

[B40] Zhang K Wong HN Song B Miller CN Scheuner D Kaufman RJ The unfolded protein response sensor IRE1alpha is required at 2 distinct steps in B cell lymphopoiesis J Clin Invest 2005 115 2 268 281 15690081 10.1172/JCI21848 PMC546421

[B41] Qiu Y Mao T Zhang Y Shao M You J Ding Q Chen Y Wu D Xie D Lin X A crucial role for RACK1 in the regulation of glucose-stimulated IRE1alpha activation in pancreatic beta cells Sci Signal 2010 3 106 ra7 10.1126/scisignal.2000514 20103773 PMC2940714

[B42] Pole JC Courtay-Cahen C Garcia MJ Blood KA Cooke SL Alsop AE Tse DM Caldas C Edwards PA High-resolution analysis of chromosome rearrangements on 8p in breast, colon and pancreatic cancer reveals a complex pattern of loss, gain and translocation Oncogene 2006 25 41 5693 5706 10.1038/sj.onc.1209570 16636668

[B43] Haverty PM Fridlyand J Li L Getz G Beroukhim R Lohr S Wu TD Cavet G Zhang Z Chant J High-resolution genomic and expression analyses of copy number alterations in breast tumors Genes Chromosomes Cancer 2008 47 6 530 542 10.1002/gcc.20558 18335499

[B44] Holland DG Burleigh A Git A Goldgraben MA Perez-Mancera PA Chin SF Hurtado A Bruna A Ali HR Greenwood W ZNF703 is a common Luminal B breast cancer oncogene that differentially regulates luminal and basal progenitors in human mammary epithelium EMBO Mol Med 2011 3 3 167 180 10.1002/emmm.201100122 21337521 PMC3395113

[B45] Sircoulomb F Nicolas N Ferrari A Finetti P Bekhouche I Rousselet E Lonigro A Adelaide J Baudelet E Esteyries S ZNF703 gene amplification at 8p12 specifies luminal B breast cancer EMBO Mol Med 2011 3 3 153 166 10.1002/emmm.201100121 21328542 PMC3395112

[B46] Chin K DeVries S Fridlyand J Spellman PT Roydasgupta R Kuo WL Lapuk A Neve RM Qian Z Ryder T Genomic and transcriptional aberrations linked to breast cancer pathophysiologies Cancer Cell 2006 10 6 529 541 10.1016/j.ccr.2006.10.009 17157792

[B47] Woods Ignatoski KM Dziubinski ML Ammerman C Ethier SP Cooperative interactions of HER-2 and HPV-16 oncoproteins in the malignant transformation of human mammary epithelial cells Neoplasia 2005 7 8 788 798 10.1593/neo.05106 16207481 PMC1501888

[B48] Behbod F Kittrell FS LaMarca H Edwards D Kerbawy S Heestand JC Young E Mukhopadhyay P Yeh HW Allred DC An intraductal human-in-mouse transplantation model mimics the subtypes of ductal carcinoma in situ Breast Cancer Res 2009 11 5 R66 10.1186/bcr2358 19735549 PMC2790841

[B49] Browman DT Hoegg MB Robbins SM The SPFH domain-containing proteins: more than lipid raft markers Trends Cell Biol 2007 17 8 394 402 10.1016/j.tcb.2007.06.005 17766116

[B50] Jo Y Sguigna PV DeBose-Boyd RA Membrane-associated ubiquitin ligase complex containing gp78 mediates sterol-accelerated degradation of 3-hydroxy-3-methylglutaryl-coenzyme A reductase J Biol Chem 2011 286 17 15022 15031 10.1074/jbc.M110.211326 21343306 PMC3083207

[B51] Shajahan AN Riggins RB Clarke R The role of X-box binding protein-1 in tumorigenicity Drug News Perspect 2009 22 5 241 246 10.1358/dnp.2009.22.5.1378631 19609461 PMC3510657

[B52] Shen X Zhang K Kaufman RJ The unfolded protein response–a stress signaling pathway of the endoplasmic reticulum J Chem Neuroanat 2004 28 1–2 79 92 15363493 10.1016/j.jchemneu.2004.02.006

[B53] Hetz C Martinon F Rodriguez D Glimcher LH The Unfolded Protein Response: integrating Stress Signals Through the Stress Sensor IRE1{alpha} Physiol Rev 2011 91 4 1219 1243 10.1152/physrev.00001.2011 22013210

[B54] Soule HD Maloney TM Wolman SR Isolation and characterization of a spontaneously immortalized human breast epithelial cell line, MCF-10 Cancer Res 1990 50 6075 86 1975513

[B55] Ethier SP Mahacek ML Gullick WJ Frank TS Weber BL Differential isolation of normal luminal mammary epithelial cells and breast cancer cells from primary and metastatic sites using selective media Cancer Res 1993 53 627 35 8425198

[B56] Ethier SP Kokeny KE Ridings JW Dilts CA erbB family receptor expression and growth regulation in a newly isolated human breast cancer cell line Cancer Res 1996 56 899 907 8631031

[B57] Forozan F Veldman R Ammerman CA Molecular cytogenetic analysis of 11 new breast cancer cell lines Br J Cancer 1999 81 1328 34 10.1038/sj.bjc.6695007 10604729 PMC2362964

[B58] Ray ME Yang ZQ Albertson D Genomic and expression analysis of the 8p11-12 amplicon in human breast cancer cell lines Cancer Res 2004 64 40 7 10.1158/0008-5472.CAN-03-1022 14729606

