# Supplementary material for: ERLIN2 promotes breast cancer cell survival by modulating endoplasmic reticulum stress pathways
Source: BMC Cancer. 2012 Jun 8;12:225. doi: 10.1186/1471-2407-12-225 (PMC3732090; doi:10.1186/1471-2407-12-225)

Table S1: Expression Levels of XBP1, ERLIN1 and ERLIN2 in Ten SUM Breast Cancer Cell Lines Using Our Affymetrix Array Database

TargetID	ProbeID	SUM-44	SUM-52	SUM-225	SUM185	SUM-190	SUM102	SUM-149	SUM-149	SUM-159	SUM-229	SUM-1315
XBP1	2450156	3.84	2.56	3.58	1.99	4.23	0.93	-0.22	0.25	1.24	0.31	0.01
XBP1	5690066	3.66	2.80	3.65	2.31	4.06	1.19	0.07	0.41	1.59	0.66	0.38
ERLIN1	6110634	-0.50	-0.24	-0.56	-0.22	-0.22	-0.01	-0.75	0.01	0.28	-0.25	0.26
ERLIN2	870646	2.46	0.13	1.24	0.11	0.38	-1.24	-0.83	-0.36	-1.12	-0.59	-1.28
ERLIN2	3130048	2.62	-0.19	1.30	-0.02	-0.12	-1.36	-0.84	-0.87	-1.00	-0.49	-1.39
ERLIN2	5570048	1.43	0.70	2.31	-0.43	0.17	-0.98	-0.75	-0.65	-0.71	-1.04	-1.29

Table S2: Expression of ERLIN2 in breast tissues: carcinomas and normal

IHC Score	HBC	Normal
0	2	10
1	2	4
2	6	3
3	13	0
4	11	0
Total	34	17

Figure S1

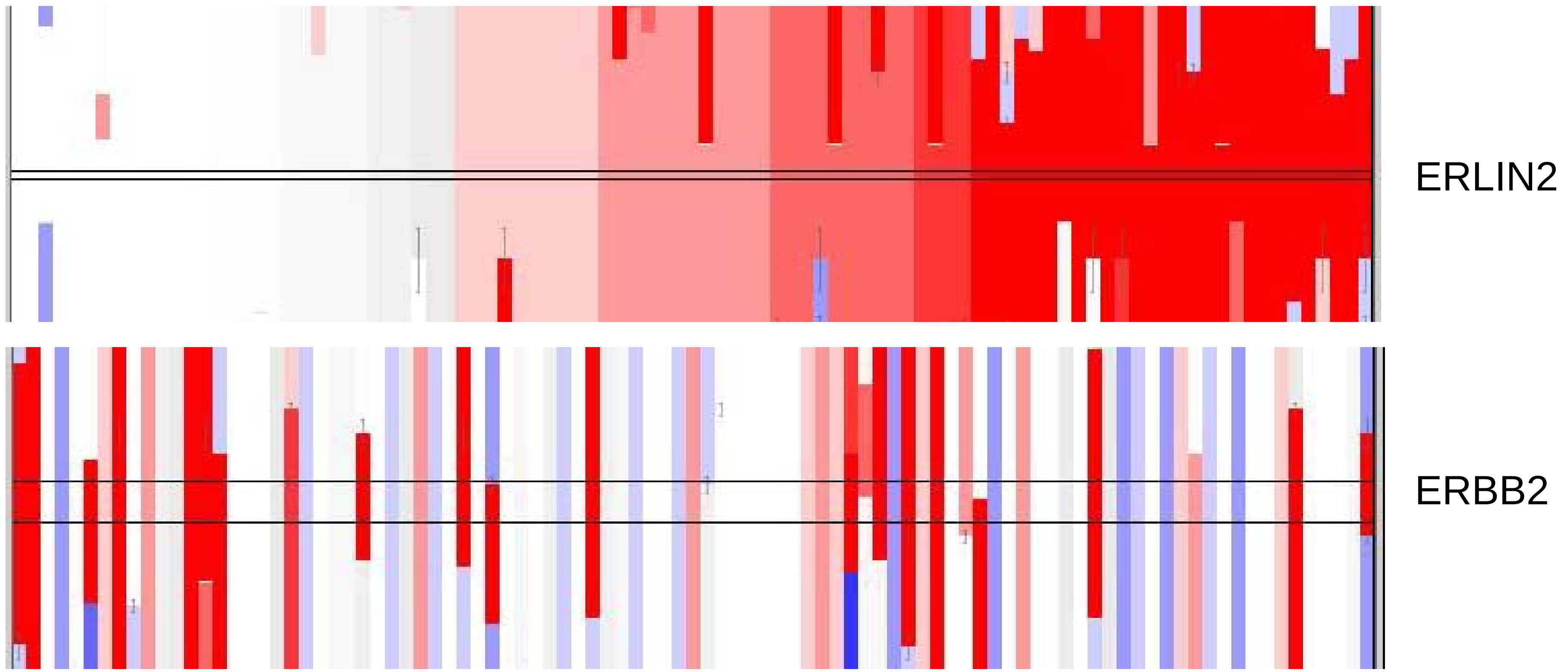

Figure S2

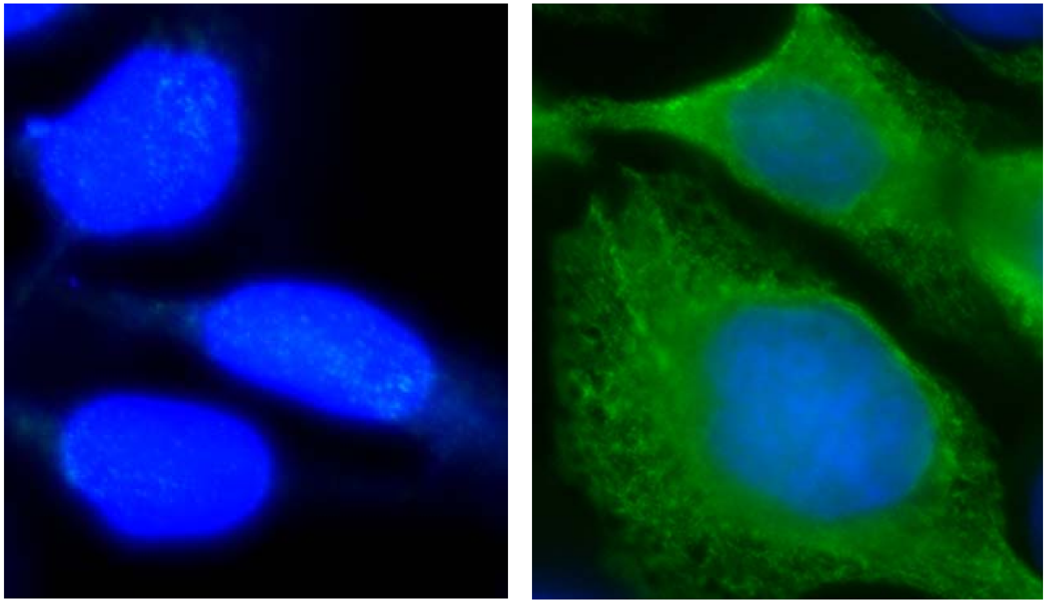

MCF10A-Ctrl MCF10A-ERLIN2

Figure S3

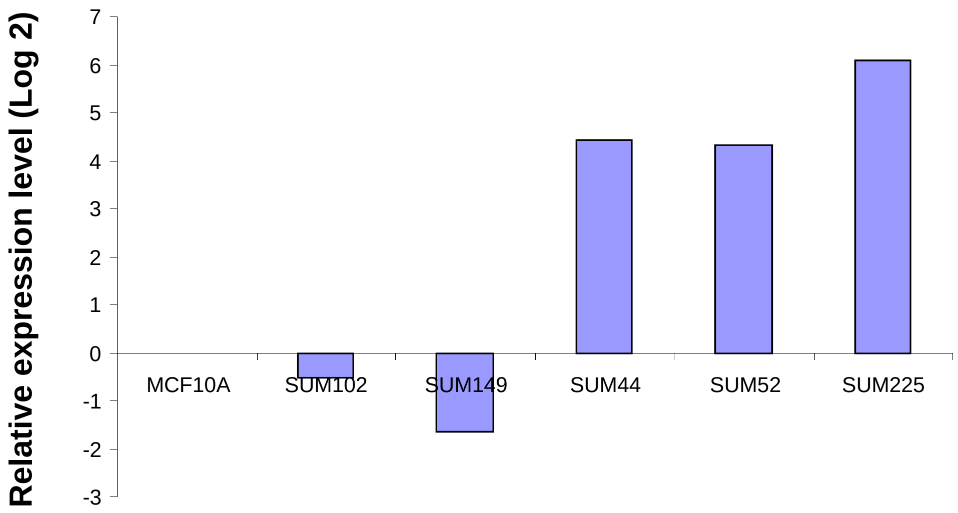

Total XBP1

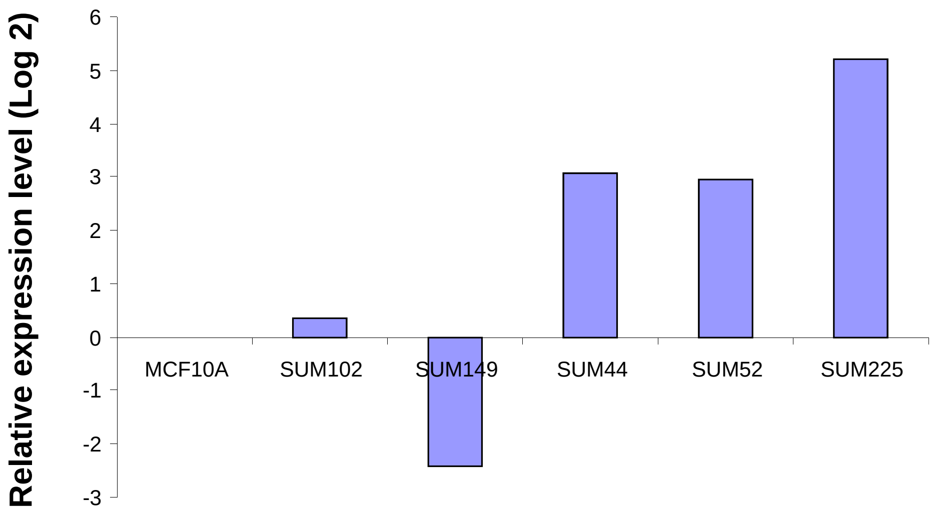

Activated XBP1

Figure S4

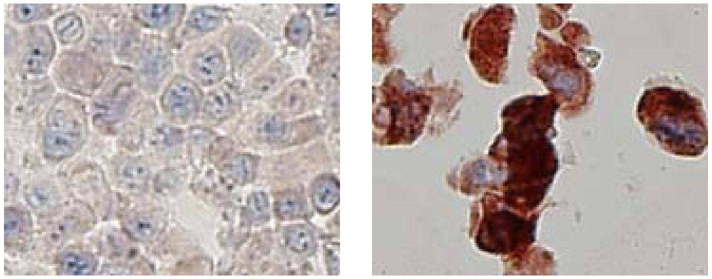

MCF10A SUM-225

Figure S5

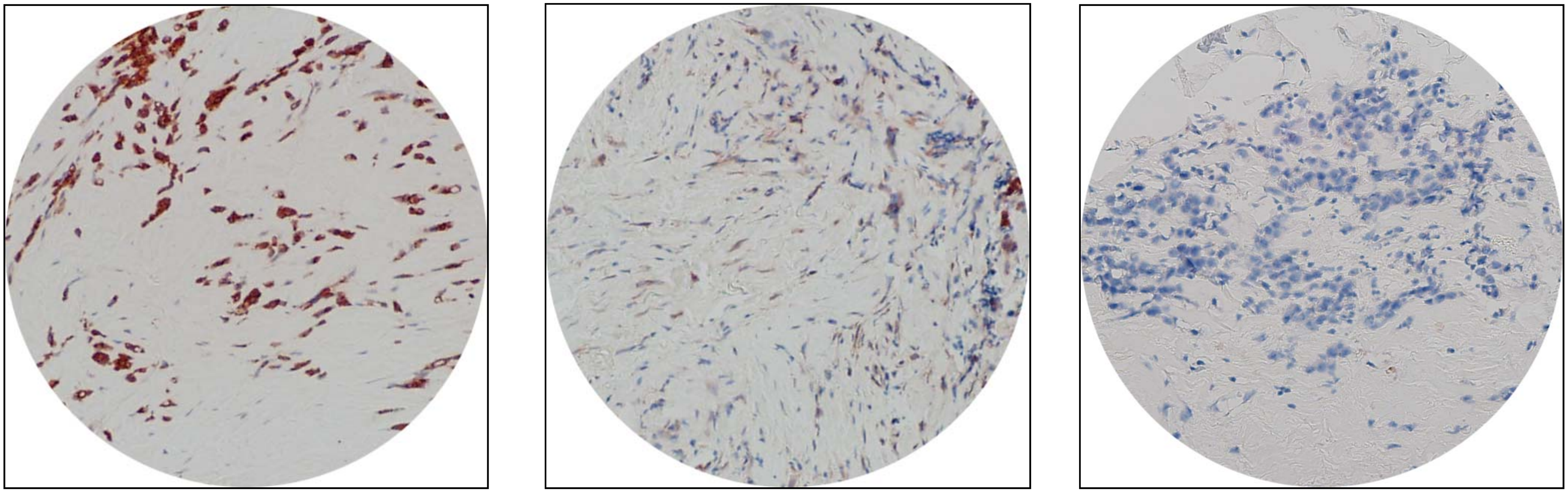

Supplement: Additional file 2: Table S1 — Expression Levels of XBP1, ERLIN1 and ERLIN2 in Ten SUM BreastCancer Cell Lines Using Our Affymetrix Array Database. Table S2: Expression of ERLIN2 in breast tissues:carcinomas and normal. Figure S1. Figure S2. Figure S3. Figure S4. Figure S5. [file 1471-2407-12-225-S2.pdf]
